# Sodium–glucose cotransporter-2 inhibitor use is associated with reduced acute kidney injury after transcatheter aortic valve implantation

**DOI:** 10.3389/fcvm.2026.1799940

**Published:** 2026-03-27

**Authors:** Berhan Keskin, Aykun Hakgor, Yerkenur Khidolda, Atakan Dursun, Aysel Akhundova, Umeyir Savur, Fatih Erkam Olgun, Ozlem Onder, Mehmet Besiroglu, Melike Zeynep Dursun, Beytullah Cakal, Haci Murat Gunes, Ibrahim Oguz Karaca, Ekrem Guler, Bilal Boztosun

**Affiliations:** Department of Cardiology, Istanbul Medipol University, Medipol Mega University Hospital, Istanbul, Türkiye

**Keywords:** acute kidney injury, contrast-inducednephropathy, propensity score matching, SGLT-2 inhibitors, transcatheter aortic valve implantation

## Abstract

**Background:**

Acute kidney injury (AKI) remains a clinically relevant complication after transcatheter aortic valve implantation (TAVI). Sodium-glucose cotransporter-2 inhibitors (SGLT-2i) have demonstrated nephroprotective effects in chronic kidney disease (CKD); however, TAVI-specific data are limited.

**Methods:**

We analyzed a single-center registry of consecutive patients who underwent transfemoral TAVI for aortic stenosis between January 2015 and December 2025. After exclusions, 532 patients were included (SGLT-2i users, *n* = 112; non-users, *n* = 420). The primary endpoint was post-procedural AKI. Secondary outcomes were need for hemodialysis and in-hospital mortality. Propensity score matching (PSM) was performed (1:1), yielding 110 matched pairs.

**Results:**

In the overall cohort, AKI occurred more frequently in SGLT-2i non-users than users (16.0% vs. 4.5%, *p* < 0.001), along with a higher requirement for hemodialysis (6.0% vs. 0.9%, *p* = 0.025). In the CKD subgroup, non-users had higher AKI (35.0% vs. 4.5%, *p* < 0.001) and hemodialysis rates (15.0% vs. 0.0%, *p* = 0.005), whereas outcomes were similar in the non-CKD subgroup. In the PSM cohort, non-users had higher AKI (20.0% vs. 4.5%, *p* < 0.001), hemodialysis (7.3% vs. 0.9%, *p* = 0.035), and in-hospital mortality (10.0% vs. 1.8%, *p* = 0.019). In the PSM CKD subgroup, non-users demonstrated markedly higher AKI (43.2% vs. 4.5%, *p* < 0.001), hemodialysis requirement (13.6% vs. 0.0%, *p* = 0.026), and in-hospital mortality (20.5% vs. 2.3%, *p* = 0.015), while non-CKD subgroup showed comparable outcomes. In multivariable analysis, SGLT-2i use independently predicted lower AKI risk in both the overall and matched cohorts.

**Conclusions:**

SGLT-2i use was associated with reduced AKI after TAVI, particularly in patients with CKD, and remained significant after propensity matching and multivariable adjustment.

## Introduction

Despite transcatheter aortic valve implantation (TAVI) being associated with lower rates of acute kidney injury (AKI) compared with surgical aortic valve replacement (1, 2), AKI remains a frequent and clinically relevant complication after TAVI and is consistently linked to adverse outcomes, including prolonged hospitalization and increased short- and long-term mortality (3, 4). Multiple patient- and procedure-related factors have been identified as predictors of AKI in this setting, such as advanced age, higher contrast volume, chronic kidney disease (CKD), anemia, and congestive heart failure (CHF) (5, 6).

Sodium-glucose cotransporter-2 inhibitors (SGLT-2i) have emerged as a cornerstone therapy for renal protection, demonstrating significant reductions in kidney disease progression and major adverse outcomes, including all-cause mortality, in patients with CKD across a broad spectrum of disease severity and irrespective of diabetes status, as shown in landmark randomized controlled trials such as DAPA-CKD, EMPA-KIDNEY, EMPAREG-OUTCOME and DECLARE–TIMI 58 (7–11). In parallel, accumulating observational evidence suggests that SGLT-2i may also confer protection against AKI in high-risk clinical scenarios, including contrast-induced nephropathy (CIN) following coronary angiography or percutaneous coronary intervention (12–16).

Patients undergoing TAVI represent a particularly vulnerable population with an intrinsically elevated baseline risk for AKI due to older age and a high prevalence of comorbid conditions such as CKD and anemia (17). However, the effect of SGLT-2i on renal outcomes, particularly AKI, following TAVI has not been adequately defined. Addressing this knowledge gap may have important implications for optimizing peri-procedural renal protection strategies in contemporary TAVI practice.

Therefore, this study aimed to evaluate the association between SGLT-2i use and the development of AKI after TAVI, and to further explore this relationship in a prespecified subgroup analysis according to baseline CKD status.

## Methods

### Study population

This study used data from a single-center registry of patients who underwent transfemoral TAVI for severe symptomatic aortic stenosis between January 2015 and December 2025.

Initially, 578 patients in the registry were screened, and the exclusion criteria were as follows:

- Cardiogenic shock during the procedure or in the early postoperative period (*n* = 7)
- Shock or death due to major structural complications, including aortic dissection, annular rupture, cardiac tamponade, coronary obstruction, etc. (*n* = 8)
- End-stage renal failure [estimated glomerular filtration rate (GFR) < 30 mL/min/1.73 m^2^ or chronic hemodialysis] (*n* = 15)
- Missing data (*n* = 11)
- Major bleeding resulting in shock (*n* = 4)
- Early post-operative mortality due to stroke (*n* = 1)

**Figure 1:**
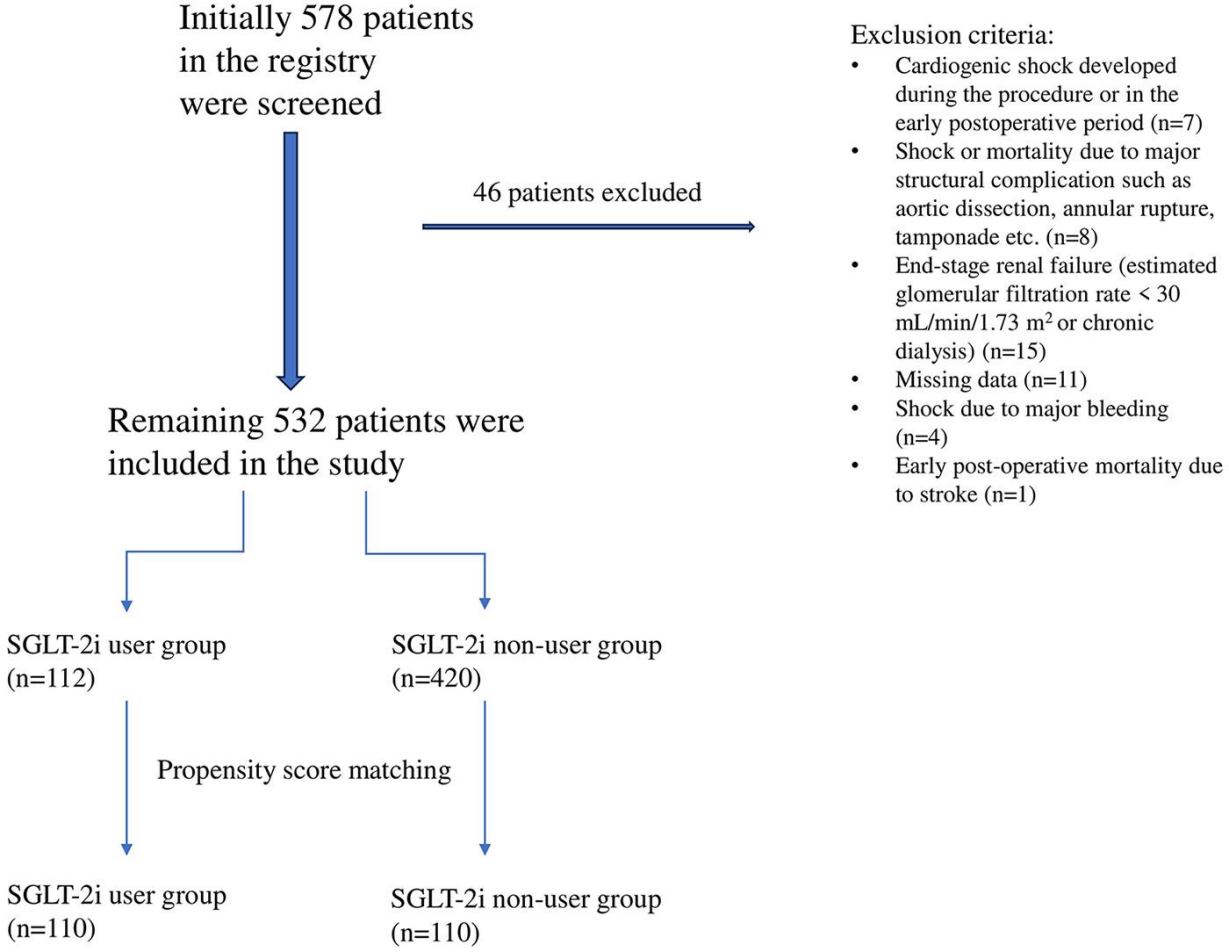
Study flow diagram.

After excluding 46 patients based on these criteria, the remaining 532 patients constituted the final study cohort, as shown in the flow diagram (Figure 1).

The study was conducted in accordance with the ethical principles of the Declaration of Helsinki and was approved by the institutional ethics committee on October 13, 2025 (approval number: 1385). Written informed consent was obtained from all participants at hospital admission and prior to invasive procedures, including consent for the scientific use of their data.

### Data collection and procedural information

Patient data on baseline characteristics, comorbidities, medications, echocardiographic evaluations, laboratory values, and procedural details were obtained from the registry database, patient medical files, and the hospital electronic health record system. Baseline clinical parameters, comorbidities, and chronic medications were recorded at hospital admission. Laboratory values were obtained at admission and monitored daily throughout the hospitalization period. Total contrast volume and in-hospital medications, including SGLT-2i, were recorded from patient files and the hospital electronic system. All patients receiving SGLT-2 inhibitors had been on chronic therapy prior to admission with either dapagliflozin 10 mg/day or empagliflozin 25 mg/day, and empagliflozin 10 mg was not used in our registry population. In our institutional protocol, SGLT-2 inhibitor therapy was not discontinued before coronary angiography or the TAVI procedure and was continued throughout the hospitalization period with routine metabolic monitoring. SGLT-2 inhibitors were not initiated *de novo* during the peri-procedural period. Furthermore, no patient in the SGLT-2i non-user group had been receiving SGLT-2 inhibitor therapy prior to admission that was subsequently discontinued during hospitalization. In accordance with institutional protocol to reduce the risk of contrast-induced nephropathy, metformin was discontinued at the time of hospital admission (≥2 days prior to contrast exposure) in all patients.

Transthoracic echocardiography was performed on the day of admission before the procedure by an experienced echocardiography specialist using a Vivid E95 system (GE Vingmed Ultrasound, Milwaukee, WI, USA). All measurements were performed in accordance with the recommendations of the European Association of Cardiovascular Imaging and the American Society of Echocardiography (18). Left ventricular ejection fraction (LVEF) was assessed using the biplane Simpson's method.

Per institutional protocol, all patients underwent diagnostic coronary angiography via radial approach prior to TAVI, typically 2 days before the valvular intervention. Decisions regarding coronary revascularization were made by the interventional cardiology team based on coronary anatomy and clinical presentation. A luminal stenosis ≥90% in vessels with a reference diameter ≥2.5 mm was considered hemodynamically significant and treated with percutaneous coronary intervention (PCI) when deemed appropriate.

All TAVI procedures were performed via transfemoral access under ultrasound and fluoroscopic guidance. The side of femoral access was determined based on pre-procedural computed tomography (CT) assessment. A 6F pigtail catheter was used for contrast injections during valve deployment. Temporary pacing was established at the beginning of the procedure via the femoral vein on the contralateral (non-access) side, and all valve implantations were performed under rapid ventricular pacing. Standard CT analyses were performed using 3mensio Structural Heart software (Pie Medical Imaging, Maastricht, the Netherlands) before the procedure, and valve size selection was based on these measurements. The choice of valve type was determined by the interventional team after comprehensive case-based evaluation.

Vascular closure was performed using Perclose ProGlide (Abbott Vascular, Santa Clara, CA, USA) and/or Angio-Seal (Terumo Corp., Tokyo, Japan) devices when feasible. Surgical repair or covered stent implantation was undertaken in cases of closure failure or major vascular complications.

### Definitions

Clinical endpoint definitions were primarily based on the Valve Academic Research Consortium-3 (VARC-3) criteria (19). Periprocedural complications, including major and minor vascular complications, were recorded according to VARC-3 definitions. In line with VARC-3, bleeding events of type ≥2 were classified as major bleeding, whereas type 1 bleeding events were classified as minor bleeding. Technical success was defined as fulfillment of all of the following criteria at the end of the TAVI procedure: absence of procedural mortality; successful vascular access; successful delivery and retrieval of the delivery system; correct valve positioning; and no requirement for surgery or further intervention due to device-related complications, major vascular complications, or major cardiac structural complications.

CKD (estimated GFR < 60 mL/min/m^2^) and AKI were defined according to the Kidney Disease Improving Global Outcomes (KDIGO) guidelines. AKI was defined as the presence of any of the following criteria after the TAVI procedure:

- An increase in serum creatinine ≥0.3 mg/dL (≥26.5 µmol/L) from baseline within 48 h
- An increase in serum creatinine to >1.5 times the baseline value within 7 days
- Urine output <0.5 mL/kg/h for at least 6 h.

### Outcomes

The primary endpoint of the study was the development of AKI following TAVI. Secondary outcomes included the requirement for hemodialysis and in-hospital mortality.

### Statistical analysis

Patients were categorized according to SGLT-2i use, and groups were compared with respect to baseline and procedural characteristics as well as post-procedural outcomes. Categorical variables were compared using the chi-square test or Fisher's exact test, as appropriate. Continuous variables were assessed for normality using visual inspection of histograms and the Kolmogorov–Smirnov test. Normally distributed variables were compared using the student's *t*-test, whereas non-normally distributed variables were compared using the Mann–Whitney *U* test. Continuous data are presented as mean ± standard deviation for normally distributed variables and as median (interquartile range) for non-normally distributed variables. Categorical variables are reported as counts and percentages. Additional comparisons were performed in subgroups stratified by CKD status.

To minimize baseline differences between the two groups, propensity score matching (PSM) was performed in the overall cohort. Propensity scores were estimated using multivariable logistic regression with SGLT-2i use as the dependent variable. The propensity score model included clinically relevant covariates selected *a priori*, including age, sex, body-mass index (BMI), CKD status, LVEF, diabetes mellitus, congestive heart failure, insulin use, and baseline creatinine. Patients were matched using nearest-neighbor 1:1 matching on the logit of the propensity score with a caliper width of 0.5 standard deviations. Matching was performed with replacement and within CKD strata (exact matching by CKD status) to ensure balance with respect to baseline renal impairment. Only matched pairs within the region of common support were retained for the matched cohort. Covariate balance was evaluated using standardized mean differences (SMDs). Following matching, baseline characteristics and outcomes were re-analyzed in the matched population, including separate analyses in CKD and non-CKD subgroups. Outcomes of unmatched and propensity-matched analyses were illustrated using bar plots showing event rates (%) for AKI and need for hemodialysis across the overall cohort and CKD strata.

To identify independent predictors of AKI following TAVI, univariate and multivariable logistic regression analyses were performed in the overall cohort. Baseline clinical characteristics, comorbidities, medications, laboratory values, echocardiographic parameters, and procedural variables were evaluated in univariate models, and variables with *p* < 0.10 were subsequently entered into the multivariable model. Separate univariate and multivariable logistic regression analyses were also conducted in the propensity-matched cohort; variables with *p* < 0.05 on univariate analysis were included in the multivariable model. Results are presented as odds ratios (ORs) with 95% confidence intervals (CIs) and were visualized using a forest plot. To assess internal validity and reduce the risk of overfitting, bootstrap resampling (500 iterations) was performed and optimism-corrected model performance metrics were reported.

A two-sided *p* value < 0.05 was considered statistically significant. All analyses were performed using Python version 3.14.0 (Python Software Foundation, Wilmington, DE, USA).

## Results

The overall study population consisted of 532 patients, including 112 patients in the SGLT-2i user group and 420 in the non-user group. Among SGLT-2i users, 86 patients (76.8%) were receiving dapagliflozin 10 mg/day, whereas 26 patients (23.2%) were receiving empagliflozin 25 mg/day. Compared with non-users, SGLT-2i users were more frequently male (62.5% vs. 47.6%, *p* = 0.007) and were younger (75.76 ± 8.54 vs. 78.22 ± 7.88 years, *p* = 0.007) (Supplementary Table S1). Regarding comorbidities and medications, the SGLT-2i group had higher prevalences of diabetes mellitus (DM) (58.0% vs. 26.0%, *p* < 0.001), congestive heart failure (CHF) (46.4% vs. 16.0%, *p* < 0.001), and insulin use (22.3% vs. 11.9%, *p* = 0.008) (Supplementary Table S1). Other baseline clinical characteristics, comorbidities, and medications were comparable between groups.

On echocardiography, SGLT-2i users had lower left ventricular ejection fraction (LVEF) (43.97 ± 13.00% vs. 52.95 ± 11.45%, *p* < 0.001), lower maximum aortic gradient (62.78 ± 23.74 vs. 72.37 ± 21.33 mmHg, *p* < 0.001), and lower tricuspid annular plane systolic excursion (TAPSE) (1.83 ± 0.31 vs. 1.95 ± 0.31 cm, *p* = 0.006). In addition, the SGLT-2i group demonstrated larger left atrial anteroposterior diameter and larger left ventricular end-diastolic and end-systolic diameters, as well as a higher frequency of low-flow low-gradient severe aortic stenosis (24.1% vs. 10.8%, *p* < 0.001) (Supplementary Table S2). Aortic valve area, pulmonary artery systolic pressure (PASP), and rates of moderate-to-severe mitral, aortic, and tricuspid regurgitation were similar between groups (Supplementary Table S2).

Laboratory analyses showed higher baseline creatinine levels in the SGLT-2i group (1.20 ± 0.53 vs. 1.11 ± 0.50 mg/dL, *p* = 0.030), whereas other laboratory parameters did not differ significantly (Supplementary Table S2). The SGLT-2i group also received a higher total contrast volume during index hospitalization (141.75 ± 82.63 vs. 123.78 ± 72.78 mL, *p* = 0.028) (Supplementary Table S2). These baseline imbalances were generally consistent across CKD subgroups.

To balance baseline differences between the groups, propensity score matching (PSM) was performed, resulting in 110 matched pairs (66 pairs without CKD and 44 pairs with CKD). Covariate balance was evaluated using standardized mean differences (SMDs). After matching, all covariates demonstrated adequate balance, with SMDs close to zero (most < 0.10), indicating good comparability between groups (Supplementary Table S3).

In the matched cohort, sex distribution (61.8% vs. 60.9%, *p* = 1.000) and age (76.01 ± 8.40 vs. 75.04 ± 10.45 years, *p* = 0.606) were comparable. Baseline clinical characteristics, comorbidities including diabetes and CHF, and medications were also well balanced between groups (Table 1). Echocardiographic parameters (including LVEF, left ventricular diameters, left atrial diameter, maximum aortic gradient, and low-flow low-gradient severe aortic stenosis) were similar (Table 2). Laboratory parameters, including baseline creatinine (1.20 ± 0.53 vs. 1.16 ± 0.48 mg/dL, *p* = 0.553), did not differ between groups (Table 2). Procedural characteristics, including valve platform and size distributions, valve-in-valve procedures, post-dilatation rates, and total contrast volumes (141.13 ± 81.49 vs. 144.90 ± 88.85 mL, *p* = 0.980), were also comparable (Table 2). Similar balance was observed within the CKD and non-CKD subgroups of the matched cohort (Table 2).

**Table 1:** Comparison of baseline characteristics, comorbidities, and medications in the PSM-cohort.

Variables	Overall patient cohort (*n* = 220)			Non-CKD subgroup (*n* = 132)			CKD subgroup (*n* = 88)		
	SGLT-2i user (*n* = 110)	SGLT-2i non-user (*n* = 110)	*p*-value	SGLT-2i user (*n* = 66)	SGLT-2i non-user (*n* = 66)	*p*-value	SGLT-2i user (*n* = 44)	SGLT-2i non-user (*n* = 44)	*p*-value
Baseline characteristics & demographics									
Age (years)	76.01 ± 8.40	75.04 ± 10.45	0.606	75.77 ± 7.37	72.74 ± 11.20	0.126	76.36 ± 9.82	78.48 ± 8.19	0.369
Male sex	68 (61.8%)	67 (60.9%)	1.000	43 (65.2%)	40 (60.6%)	0.719	25 (56.8%)	27 (61.4%)	0.829
BMI (kg/m^2^)	27.16 ± 5.28	27.45 ± 5.32	0.656	26.60 ± 5.15	27.85 ± 5.00	0.054	28.06 ± 5.41	26.84 ± 5.78	0.118
Comorbidities									
Hypertension	89 (80.9%)	94 (85.5%)	0.471	57 (86.4%)	62 (93.9%)	0.242	32 (72.7%)	32 (72.7%)	1.000
Diabetes mellitus	63 (57.3%)	57 (51.8%)	0.498	37 (56.1%)	30 (45.5%)	0.296	26 (59.1%)	27 (61.4%)	1.000
Atrial fibrillation	45 (40.9%)	35 (31.8%)	0.207	23 (34.8%)	22 (33.3%)	1.000	22 (50.0%)	13 (29.5%)	0.081
Congestive heart failure	50 (45.5%)	48 (43.6%)	0.892	29 (43.9%)	22 (33.3%)	0.283	21 (47.7%)	26 (59.1%)	0.393
COPD	61 (55.5%)	55 (50.0%)	0.500	33 (50.0%)	19 (28.8%)	0.020	28 (63.6%)	36 (81.8%)	0.093
Previous stroke	2 (1.8%)	5 (4.5%)	0.446	2 (3.0%)	0 (0.0%)	0.496	0 (0.0%)	5 (11.4%)	0.055
Chronic kidney disease	44 (40.0%)	44 (40.0%)	1.000	N/A	N/A	N/A	N/A	N/A	N/A
Coronary artery disease	73 (66.4%)	68 (61.8%)	0.574	43 (65.2%)	39 (59.1%)	0.591	30 (68.2%)	29 (65.9%)	1.000
Previous CABG	22 (20.0%)	18 (16.4%)	0.600	18 (27.3%)	10 (15.2%)	0.135	4 (9.1%)	8 (18.2%)	0.352
Pre-operative pacemaker	3 (2.7%)	4 (3.6%)	1.000	2 (3.0%)	4 (6.1%)	0.680	1 (2.3%)	0 (0.0%)	1.000
Medications									
ACEi/ARB	61 (55.5%)	69 (62.7%)	0.337	41 (62.1%)	47 (71.2%)	0.356	20 (45.5%)	22 (50.0%)	0.831
β-blocker	102 (92.7%)	92 (83.6%)	0.060	61 (92.4%)	53 (80.3%)	0.074	41 (93.2%)	39 (88.6%)	0.713
Statins	78 (70.9%)	76 (69.1%)	0.883	47 (71.2%)	47 (71.2%)	1.000	31 (70.5%)	29 (65.9%)	0.819
Insulin	25 (22.7%)	24 (21.8%)	1.000	10 (15.2%)	11 (16.7%)	1.000	15 (34.1%)	13 (29.5%)	0.819
Anticoagulation	40 (36.4%)	32 (29.1%)	0.315	20 (30.3%)	21 (31.8%)	1.000	20 (45.5%)	11 (25.0%)	0.073

CKD, chronic kidney disease; SGLT-2i, sodium-glucose cotransporter-2 inhibitor; BMI, body-mass index; COPD, chronic obstructive pulmonary disease; CABG, coronary artery bypass grafting; ACEi, angiotensin-converting enzyme inhibitor; ARB, angiotensin receptor blockers.

**Table 2:** Comparison of pre-procedural echocardiographic parameters, laboratory values, and procedural data in the PSM-cohort.

Variables	Overall patient cohort (*n* = 220)			Non-CKD subgroup (*n* = 132)			CKD subgroup (*n* = 88)		
	SGLT-2i user (*n* = 110)	SGLT-2i non-user (*n* = 110)	*p*-value	SGLT-2i user (*n* = 66)	SGLT-2i non-user (*n* = 66)	*p*-value	SGLT-2i user (*n* = 44)	SGLT-2i non-user (*n* = 44)	*p*-value
Pre-procedural echocardiographic parameters									
LVEF (%)	44.20 ± 13.00	43.14 ± 14.06	0.611	45.05 ± 12.24	46.11 ± 12.97	0.543	38.75 ± 14.59	42.93 ± 14.10	0.177
LVEDD (cm)	5.26 ± 0.66	5.11 ± 0.84	0.067	5.19 ± 0.56	5.00 ± 0.79	0.056	5.32 ± 0.91	5.36 ± 0.77	0.663
LVESD (cm)	3.79 ± 0.79	3.75 ± 0.99	0.246	3.68 ± 0.71	3.60 ± 0.84	0.269	4.01 ± 1.19	3.94 ± 0.87	0.827
Maximum aortic gradient (mmHg)	62.96 ± 23.78	69.21 ± 26.98	0.392	67.19 ± 23.51	73.10 ± 31.64	0.772	56.52 ± 22.98	63.51 ± 16.92	0.108
Aortic valve area (cm^2^)	0.74 ± 0.16	0.74 ± 0.18	0.930	0.73 ± 0.16	0.74 ± 0.19	0.714	0.76 ± 0.16	0.75 ± 0.17	0.355
Low-flow low-gradient AS	26 (23.6%)	29 (26.6%)	0.726	10 (15.2%)	14 (21.5%)	0.375	16 (36.4%)	15 (34.1%)	1.000
TAPSE (cm)	1.83 ± 0.31	1.91 ± 0.42	0.466	1.84 ± 0.33	2.04 ± 0.45	0.044	1.82 ± 0.29	1.77 ± 0.34	0.459
PASP (mmHg)	49.93 ± 14.69	49.77 ± 13.15	0.793	51.20 ± 15.16	46.83 ± 12.55	0.123	47.95 ± 13.88	54.55 ± 12.83	0.027
Left atrial diameter (cm)	4.50 ± 0.57	4.48 ± 0.63	0.583	4.50 ± 0.57	4.39 ± 0.55	0.269	4.51 ± 0.58	4.60 ± 0.72	0.697
Aortic root diameter (cm)	2.83 ± 0.51	2.72 ± 0.52	0.246	2.81 ± 0.50	2.72 ± 0.51	0.693	2.85 ± 0.52	2.72 ± 0.54	0.148
Moderate-to-severe mitral regurgitation	70 (63.6%)	80 (72.7%)	0.193	38 (57.6%)	53 (80.3%)	0.008	32 (72.7%)	27 (61.4%)	0.364
Moderate-to-severe tricuspid regurgitation	70 (63.6%)	71 (64.5%)	1.000	42 (63.6%)	40 (60.6%)	0.858	28 (63.6%)	31 (70.5%)	0.650
Moderate-to-severe aortic regurgitation	49 (44.5%)	59 (53.6%)	0.225	28 (42.4%)	36 (54.5%)	0.223	21 (47.7%)	23 (52.3%)	0.831
Laboratory values									
Pre-op hemoglobin (g/dL)	11.71 ± 1.68	11.63 ± 1.86	0.451	11.85 ± 1.72	12.17 ± 1.91	0.558	11.51 ± 1.63	10.81 ± 1.47	0.037
Pre-op creatinine (mg/dL)	1.20 ± 0.53	1.16 ± 0.48	0.553	0.91 ± 0.17	0.88 ± 0.19	0.224	1.64 ± 0.58	1.59 ± 0.48	0.867
Post-op hemoglobin (g/dL)	10.35 ± 1.58	10.01 ± 1.60	0.069	10.38 ± 1.48	10.34 ± 1.65	0.695	10.30 ± 1.74	9.52 ± 1.40	0.024
Leucocytes (cell count/L)	(7.85 ± 2.27) × 10^3^	(7.83 ± 2.27) × 10^3^	0.714	(7.60 ± 2.17) × 10^3^	(7.33 ± 1.86) × 10^3^	0.444	(8.23 ± 2.38) × 10^3^	(8.59 ± 2.64) × 10^3^	0.625
Platelets (cell count/mcL)	(221.44 ± 64.53) × 10^3^	(233.50 ± 83.19) × 10^3^	0.370	(220.64 ± 64.30) ×10^3^	(240.16 ± 70.30) × 10^3^	0.051	(222.64 ± 65.61) × 10^3^	(223.44 ± 99.63) × 10^3^	0.302
Sodium (mEq/L)	137.34 ± 3.45	138.30 ± 3.28	0.076	137.77 ± 3.39	138.00 ± 2.65	0.922	136.70 ± 3.47	138.78 ± 4.10	0.015
Potassium (mEq/L)	4.35 ± 0.52	4.36 ± 0.48	0.947	4.34 ± 0.43	4.29 ± 0.40	0.507	4.37 ± 0.63	4.47 ± 0.57	0.487
AST (IU/L)	37.74 ± 78.99	26.58 ± 15.43	0.206	26.58 ± 27.26	25.49 ± 10.39	0.185	54.63 ± 119.55	28.31 ± 21.18	0.806
TSH (mIU/L)	2.38 ± 2.66	2.23 ± 2.03	0.871	2.56 ± 3.17	1.93 ± 1.95	0.474	2.09 ± 1.46	2.80 ± 2.11	0.280
Procedural data									
Self-expanding platform use	82 (74.5%)	73 (66.4%)	0.237	49 (74.2%)	49 (74.2%)	1.000	33 (75.0%)	24 (54.5%)	0.073
Valve-in-valve procedure	8 (7.3%)	7 (6.4%)	1.000	7 (10.6%)	4 (6.1%)	0.531	1 (2.3%)	3 (6.8%)	0.616
Post-dilatation	30 (27.3%)	31 (28.2%)	1.000	18 (27.3%)	21 (31.8%)	0.703	12 (27.3%)	10 (22.7%)	0.806
Valve size (mm)	29.03 ± 3.63	28.33 ± 3.47	0.226	29.09 ± 3.76	28.57 ± 3.25	0.497	28.94 ± 3.46	27.98 ± 3.79	0.237
Total contrast volume (mL)	141.13 ± 81.49	144.90 ± 88.85	0.980	146.86 ± 78.05	136.23 ± 77.51	0.330	132.26 ± 86.74	157.80 ± 103.14	0.192

CKD, chronic kidney disease; SGLT-2i, sodium-glucose cotransporter-2 inhibitor; LVEF, left ventricular ejection fraction; LVEDD, left ventricular end-diastolic diameter; LVESD, left ventricular end-systolic diameter; AS, aortic stenosis; TAPSE, tricuspid annular plane systolic excursion; PASP, pulmonary artery systolic pressure; AST, aspartate aminotransferase; TSH, thyroid-stimulating hormone.

In the overall cohort (*n* = 532), procedural complication rates, including structural complications, vascular complications, bleeding events, new permanent pacemaker implantation, and moderate-to-severe paravalvular leak (PVL), were comparable between SGLT-2i users and non-users. Technical success rates were also similar across the overall population as well as within CKD and non-CKD subgroups (Supplementary Table S4). No cases of euglycemic ketoacidosis were observed among patients receiving SGLT-2 inhibitors during the peri-procedural hospitalization period.

**Figure 2:**
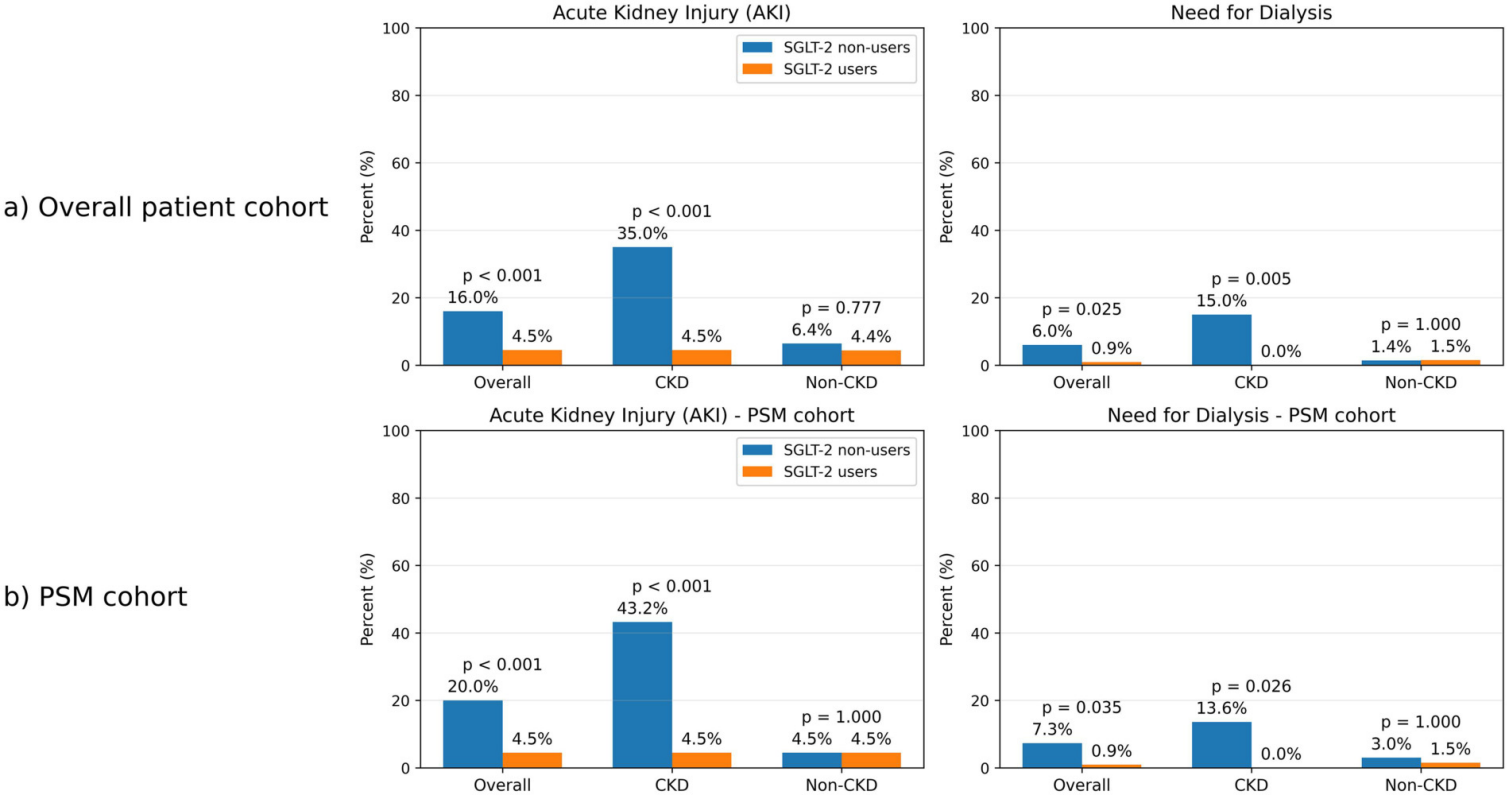
Acute kidney injury and need for dialysis in overall and propensity-matched cohorts. **(a)** Acute kidney injury and need for dialysis in overall cohort. **(b)** Acute kidney injury and need for dialysis in propensity-matched cohort.

In the overall cohort (*n* = 532), the primary outcome, AKI, occurred more frequently among SGLT-2i non-users (16.0% vs. 4.5%, *p* < 0.001), along with a higher requirement for hemodialysis (6.0% vs. 0.9%, *p* = 0.025) (Supplementary Table S4, Figure 2a). In the non-CKD subgroup (*n* = 384), AKI rates (6.4% vs. 4.4%, *p* = 0.777) and need for hemodialysis (1.4% vs. 1.5%, *p* = 1.000) were similar between groups. In contrast, in the CKD subgroup (*n* = 184), SGLT-2i non-users demonstrated substantially higher rates of AKI (35.0% vs. 4.5%, *p* < 0.001) and hemodialysis requirement (15.0% vs. 0.0%, *p* = 0.005) (Supplementary Table S4, Figure 2a). In-hospital mortality and length of hospitalization did not differ significantly between groups in the overall cohort or across CKD strata (Supplementary Table S4).

In the matched cohort (*n* = 220), procedural complications and technical success were generally similar between groups. However, the rate of new permanent pacemaker implantation was higher among SGLT-2i users (7.3% vs. 0.9%, *p* = 0.035) (Table 3). Within subgroup analyses, complication and technical success rates remained comparable in both CKD (*n* = 88) and non-CKD (*n* = 132) subgroups. An exception was a higher rate of minor bleeding among SGLT-2i users in the non-CKD subgroup (19.7% vs. 1.5%, *p* = 0.001) (Table 3).

**Table 3:** Procedural and study outcomes in PSM-cohort.

Variables	Overall patient cohort (*n* = 220)			Non-CKD subgroup (*n* = 132)			CKD subgroup (*n* = 88)		
	SGLT-2i user (*n* = 110)	SGLT-2i non-user (*n* = 110)	*p*-value	SGLT-2i user (*n* = 66)	SGLT-2i non-user (*n* = 66)	*p*-value	SGLT-2i user (*n* = 44)	SGLT-2i non-user (*n* = 44)	*p*-value
Procedural Complications & Outcomes									
Myocardial infarction/Coronary obstruction	1 (0.9%)	0 (0.0%)	1.000	1 (1.5%)	0 (0.0%)	1.000	0 (0.0%)	0 (0.0%)	1.000
Stroke	0 (0.0%)	1 (0.9%)	1.000	0 (0.0%)	0 (0.0%)	1.000	0 (0.0%)	1 (2.3%)	1.000
LV rupture/Cardiac tamponade	2 (1.8%)	0 (0.0%)	0.498	1 (1.5%)	0 (0.0%)	1.000	1 (2.3%)	0 (0.0%)	1.000
Major vascular complication	10 (9.1%)	9 (8.2%)	1.000	8 (12.1%)	3 (4.5%)	0.206	2 (4.5%)	6 (13.6%)	0.266
Minor vascular complication	16 (14.5%)	6 (5.5%)	0.041	11 (16.7%)	4 (6.1%)	0.097	5 (11.4%)	2 (4.5%)	0.434
Need for surgery due to peripheral complication	6 (5.5%)	3 (2.7%)	0.499	5 (7.6%)	3 (4.5%)	0.718	1 (2.3%)	0 (0.0%)	1.000
Major bleeding	12 (10.9%)	8 (7.3%)	0.483	8 (12.1%)	4 (6.1%)	0.365	4 (9.1%)	4 (9.1%)	1.000
Minor bleeding	19 (17.3%)	11 (10.0%)	0.168	13 (19.7%)	1 (1.5%)	0.001	6 (13.6%)	10 (22.7%)	0.408
Device embolization	1 (0.9%)	2 (1.8%)	1.000	0 (0.0%)	2 (3.0%)	0.496	1 (2.3%)	0 (0.0%)	1.000
New permanent pacemaker	8 (7.3%)	1 (0.9%)	0.035	3 (4.5%)	0 (0.0%)	0.244	5 (11.4%)	1 (2.3%)	0.202
Technical success	92 (83.6%)	98 (89.1%)	0.326	54 (81.8%)	60 (90.9%)	0.205	38 (86.4%)	38 (86.4%)	1.000
Moderate-to-severe paravalvular regurgitation	5 (4.7%)	2 (2.0%)	0.448	3 (4.6%)	0 (0.0%)	0.244	2 (4.8%)	2 (5.7%)	1.000
In-hospital Outcomes									
Hospitalization duration (days)	5.47 ± 2.96	5.86 ± 3.94	0.916	4.74 ± 2.28	5.14 ± 3.24	0.785	6.57 ± 3.51	6.95 ± 4.64	0.840
In-hospital mortality	2 (1.8%)	11 (10.0%)	0.019	1 (1.5%)	2 (3.0%)	1.000	1 (2.3%)	9 (20.5%)	0.015
Study Outcomes									
Post-procedural acute kidney injury	5 (4.5%)	22 (20.0%)	<0.001	3 (4.5%)	3 (4.5%)	1.000	2 (4.5%)	19 (43.2%)	<0.001
Need for hemodialysis	1 (0.9%)	8 (7.3%)	0.035	1 (1.5%)	2 (3.0%)	1.000	0 (0.0%)	6 (13.6%)	0.026

CKD, chronic kidney disease; SGLT-2i, sodium-glucose cotransporter-2 inhibitor; LV, left ventricle.

In the propensity-matched cohort, AKI was observed more frequently among SGLT-2i non-users (20.0% vs. 4.5%, *p* < 0.001), accompanied by higher hemodialysis requirement (7.3% vs. 0.9%, *p* = 0.035) and higher in-hospital mortality (10.0% vs. 1.8%, *p* = 0.019) (Table 3, Figure 2b). In the non-CKD subgroup, AKI (4.5% vs. 4.5%, *p* = 1.000), hemodialysis (3.0% vs. 1.5%, *p* = 1.000), and in-hospital mortality (3.0% vs. 1.5%, *p* = 1.000) were similar between SGLT-2i users and non-users (Table 3, Figure 2b). In contrast, among patients with CKD, SGLT-2i non-users showed markedly higher rates of AKI (43.2% vs. 4.5%, *p* < 0.001), need for hemodialysis (13.6% vs. 0.0%, *p* = 0.026), and in-hospital mortality (20.5% vs. 2.3%, *p* = 0.015) (Table 3, Figure 2b).

**Figure 3:**
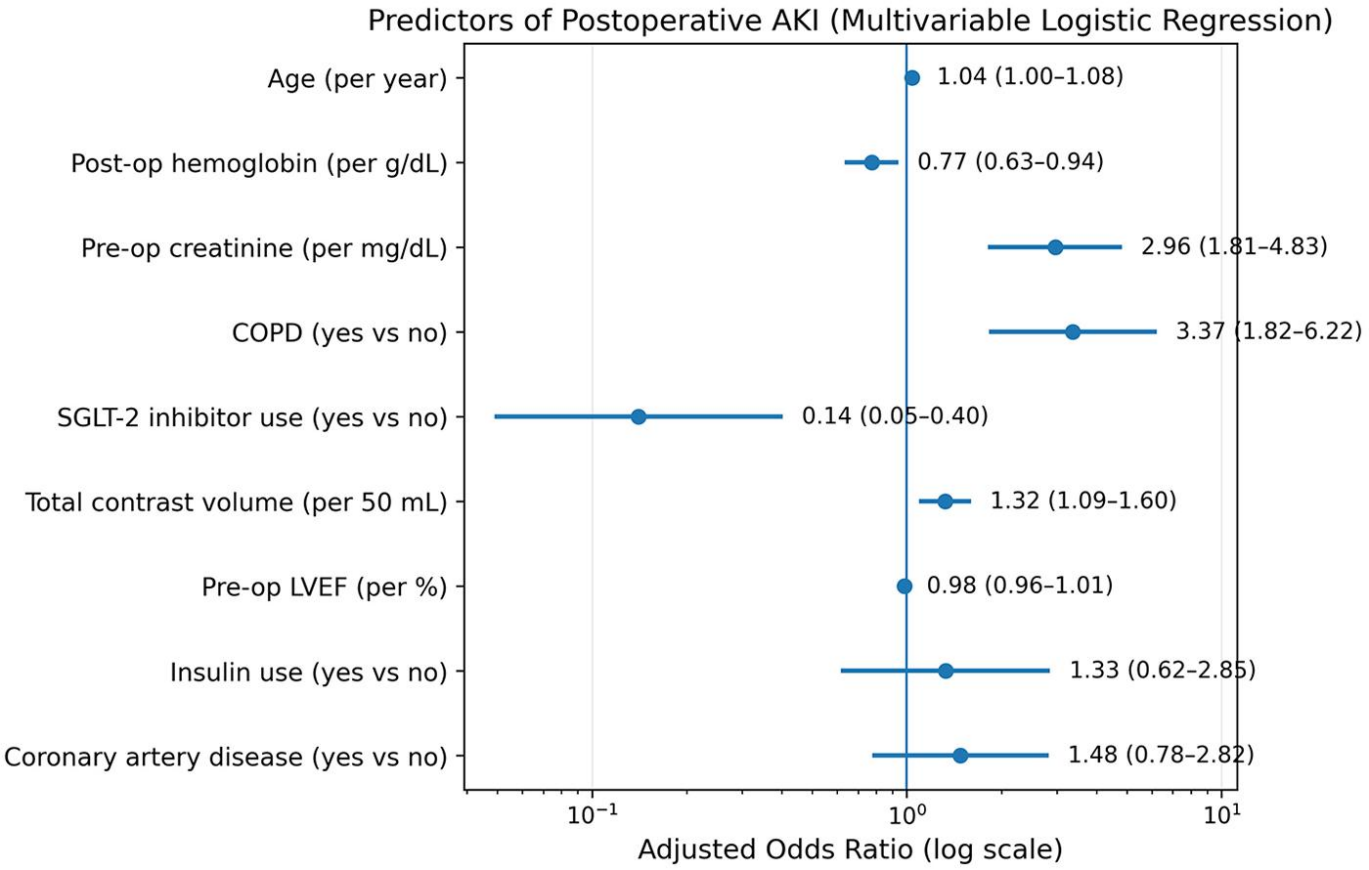
Independent predictors of postoperative acute kidney injury in the overall cohort (forest plot).

In the overall cohort (*n* = 532), univariate and multivariable logistic regression analyses were performed to identify predictors of AKI. Variables significantly associated with AKI included age, postoperative hemoglobin, baseline creatinine, chronic obstructive pulmonary disease (COPD), CKD, SGLT-2i use, preoperative hemoglobin, contrast volume, coronary artery disease, baseline LVEF, and insulin use (Table 4). To reduce collinearity, CKD and preoperative hemoglobin were not included in the multivariable model. In multivariable logistic regression analysis, postoperative hemoglobin (OR 0.733, 95% CI 0.635–0.941, *p* = 0.010), baseline creatinine (OR 2.958, 95% CI 1.812–4.829, *p* < 0.001), COPD (OR 3.368, 95% CI 1.823–6.223, *p* < 0.001), SGLT-2i use (OR 0.141, 95% CI 0.049–0.403, *p* < 0.001), and contrast volume (OR 1.322, 95% CI 1.040–1.598, *p* = 0.004) independently predicted AKI (Table 4, Figure 3). To assess model stability and potential overfitting, bootstrap resampling (500 iterations) was performed. The model demonstrated minimal optimism in discrimination (mean optimism in AUC 0.023), with an optimism-corrected AUC of 0.816. The optimism-corrected Brier score was 0.100, indicating good overall performance.

**Table 4:** Univariate and multivariable logistic regression analyses for the prediction of acute kidney injury in the overall patient cohort.

Univariate analysis				Multivariable analysis		
Predictor	Odds ratio	95% CI	*p*-value	Odds ratio	95% CI	*p*-value
Age (per 1 year)	1.064	1.026–1.104	<0.001	1.040	0.997–1.084	0.069
Post-op hemoglobin (per 1 g/dL)	0.704	0.591–0.838	<0.001	0.773	0.635–0.941	0.010
Pre-op creatinine (per 1 mg/dL)	3.461	2.251–5.323	<0.001	2.958	1.812–4.829	<0.001
COPD	3.471	2.007–6.003	<0.001	3.368	1.823–6.223	<0.001
Chronic kidney disease	5.971	3.456–10.315	<0.001			
SGLT-2i use	0.246	0.097–0.627	0.003	0.141	0.049–0.403	<0.001
Pre-op hemoglobin (per 1 g/dL)	0.804	0.692–0.934	0.004			
Contrast volume (per 50 mL)	1.227	1.057–1.424	0.007	1.322	1.04–1.598	0.004
Coronary artery disease	1.745	1.000–3.044	0.050	1.482	0.777–2.825	0.232
Pre-op LVEF (per 1%)	0.983	0.964–1.002	0.080	0.981	0.957–1.007	0.145
Insulin use	1.754	0.934–3.295	0.080	1.327	0.617–2.853	0.469
Major vascular complication	1.790	0.820–3.908	0.144			
Pre-op PASP (per 1 mmHg)	1.011	0.993–1.029	0.229			
Diabetes mellitus	1.369	0.819–2.286	0.230			
Low-flow low-gradient AS	1.333	0.678–2.623	0.405			
Pre-op maximum aortic gradient (per 1 mmHg)	0.995	0.984–1.007	0.439			
Major bleeding	1.277	0.573–2.848	0.550			
ACEi/ARB use	0.864	0.524–1.425	0.567			
New permanent pacemaker	1.299	0.481–3.509	0.607			
Moderate-to-severe PVL	1.294	0.429–3.901	0.647			
Need for surgery due to peripheral complication	1.290	0.364–4.571	0.693			
Statin use	0.932	0.558–1.558	0.789			
Hypertension	1.090	0.516–2.304	0.820			
Female sex	1.035	0.630–1.702	0.891			
Body-mass index (per 1 kg/m^2^)	1.003	0.953–1.055	0.916			

CI, confidence interval; SGLT-2i, sodium-glucose cotransporter-2 inhibitor; COPD, chronic obstructive pulmonary disease; ACEi, angiotensin-converting enzyme inhibitor; ARB, angiotensin receptor blockers; LVEF, left ventricular ejection fraction; PVL, paravalvular leak; AS, aortic stenosis; PASP, pulmonary artery systolic pressure.

**Figure 4:**
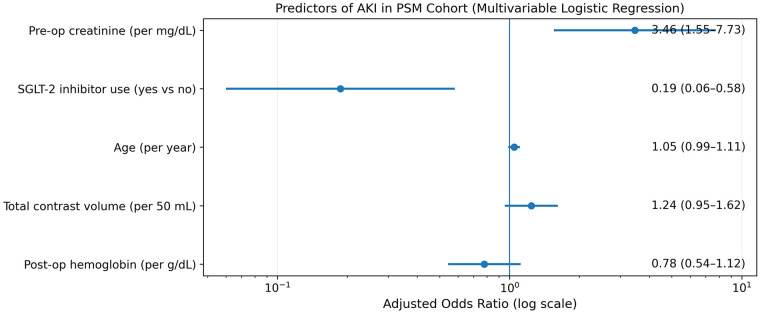
Independent predictors of acute kidney injury in the propensity score–matched cohort (forest plot).

In the propensity-matched cohort, univariate and multivariable logistic regression analyses were also conducted. On univariate analysis, age, postoperative hemoglobin, baseline creatinine, COPD, CKD, SGLT-2i use, preoperative hemoglobin, and contrast volume were associated with AKI. To reduce collinearity and overfitting risk, COPD, CKD, and preoperative hemoglobin were excluded from the final multivariable model. In the multivariable analysis, baseline creatinine (OR 3.464, 95% CI 1.553–7.728, *p* = 0.002) and SGLT-2i use (OR 0.187, 95% CI 0.060–0.580, *p* = 0.004) remained independent predictors of AKI (Table 5, Figure 4). Bootstrap internal validation (500 resamples) showed minimal optimism (AUC optimism 0.026) and preserved discrimination after correction (optimism-corrected AUC 0.802). Calibration was acceptable (slope 0.887; intercept −0.142), supporting the robustness of the multivariable model.

**Table 5:** Univariate and multivariable logistic regression analyses for the prediction of acute kidney İnjury in the PSM-cohort.

Univariate analysis				Multivariable analysis		
Predictor	Odds ratio	95% CI	*p*-value	Odds ratio	95% CI	*p*-value
Age (per 1 year)	1.075	1.016–1.137	0.013	1.046	0.988–1.109	0.124
Post-op hemoglobin (per 1 g/dL)	0.588	0.424–0.816	0.001	0.779	0.543–1.116	0.173
Pre-op creatinine (per 1 mg/dL)	2.996	1.510–5.943	0.002	3.464	1.553–7.728	0.002
COPD	4.634	1.686–12.739	0.003			
Chronic kidney disease	6.582	2.534–17.096	<0.001			
SGLT-2i use	0.190	0.069–0.524	0.001	0.187	0.060–0.580	0.004
Pre-op hemoglobin (per 1 g/dL)	0.808	0.634–1.029	0.084			
Contrast volume (per 50 mL)	1.282	1.039–1.580	0.020	1.241	0.953–1.615	0.109
Coronary artery disease	2.736	0.993–7.538	0.052			
Pre-op LVEF (per 1%)	0.973	0.944–1.003	0.080			
Insulin use	2.323	0.986–5.470	0.054			
Major vascular complication	1.383	0.375–5.098	0.626			
Pre-op PASP (per 1 mmHg)	1.022	0.993–1.053	0.135			
Diabetes mellitus	2.163	0.904–5.179	0.083			
Low-flow low-gradient AS	1.598	0.672–3.799	0.289			
Pre-op maximum aortic gradient (per 1 mmHg)	0.991	0.974–1.009	0.333			
Major bleeding	0.352	0.045–2.743	0.319			
ACEi/ARB use	1.008	0.444–2.288	0.985			
New permanent pacemaker	0.889	0.107–7.403	0.914			
Moderate-to-severe PVL	0.452	0.025–8.140	0.601			
Need for surgery due to peripheral complication	0.353	0.020–6.240	0.605			
Statin use	1.579	0.606–4.112	0.350			
Hypertension	0.875	0.308–2.480	0.801			
Female sex	1.561	0.695–3.506	0.281			
Body-mass index (per 1 kg/m^2^)	0.980	0.904–1.062	0.619			

CI, confidence interval; SGLT-2i, sodium-glucose cotransporter-2 inhibitor; COPD, chronic obstructive pulmonary disease; ACEi, angiotensin-converting enzyme inhibitor; ARB, angiotensin receptor blockers; LVEF, left ventricular ejection fraction; PVL, paravalvular leak; AS, aortic stenosis; PASP, pulmonary artery systolic pressure.

## Discussion

AKI after TAVI is a multifactorial complication with several interrelated mechanisms. First, contrast exposure may induce renal ischemia, reactive oxygen species (ROS) generation, and direct cytotoxic injury to tubular epithelial cells (20). Beyond CIN, additional TAVI-specific contributors, including cholesterol plaque embolization, hypoperfusion related to rapid pacing and periprocedural hemodynamic fluctuations, and clinically significant PVL, may also increase the risk of AKI (4). Moreover, patients undergoing TAVI generally carry a high baseline susceptibility to AKI because of advanced age and frequent coexistence of comorbidities such as anemia, CKD, and CHF (17). In our cohort, the prevalence of CKD was 34.5% and CHF was 22.4%, highlighting the substantial baseline risk for AKI in this real-world population. Therefore, generating TAVI-specific evidence regarding the potential role of SGLT-2i in reducing AKI risk is clinically important.

Several mechanisms may explain the potential protective effect of SGLT-2i against AKI. By increasing sodium delivery to macula densa and enhancing tubulo-glomerular feedback, SGLT-2i promote afferent arteriolar vasoconstriction and reduce glomerular hyperfiltration, thereby decreasing renal workload and oxygen consumption and lowering susceptibility to ischemic injury (3, 20). Beyond these hemodynamic effects, SGLT-2i exert anti-inflammatory and antioxidant actions that may mitigate tubular injury and fibrosis. SGLT-2i may also attenuate activation of the renin–angiotensin–aldosterone system and sympathetic pathways (20). In addition, increased urinary glucose excretion may reduce renal glucotoxicity and ROS-related tubular damage. Collectively, these mechanisms support the nephroprotective role of SGLT-2i, as demonstrated in CKD populations (7–11), and provide a biologically plausible rationale for their potential to reduce AKI risk, particularly in vulnerable patients such as those undergoing TAVI.

Previous studies have reported a protective association between SGLT-2i therapy and reduced CIN in patients undergoing coronary angiography or percutaneous coronary intervention (12–16). However, most of the available evidence has been derived from observational studies involving largely unmatched populations. Importantly, the TAVI setting differs substantially from coronary procedures because of unique procedural physiology and distinct patient characteristics, emphasizing the need for dedicated TAVI-specific data. As AKI has been consistently identified as a key predictor of in-hospital mortality and adverse outcomes after TAVI (21), strategies that reduce AKI may have meaningful clinical impact. Notably, SGLT-2i users frequently represent a higher-risk baseline population due to higher rates of CHF and diabetes mellitus (DM), and therefore, adjusted and matched comparisons are essential to support more reliable conclusions.

In the present study, SGLT-2i use was associated with a lower incidence of AKI and a reduced requirement for hemodialysis in both the overall cohort and the propensity score–matched cohort. A key finding was that this protective association was most pronounced among patients with CKD, whereas AKI rates were comparable between SGLT-2i users and non-users in the non-CKD subgroup. This observation suggests that the nephroprotective effects of SGLT-2i may be particularly relevant in CKD, a population with increased vulnerability to peri-procedural renal injury. Another important observation was that SGLT-2i use was not associated with lower in-hospital mortality in the unmatched cohort, likely reflecting baseline imbalances and higher comorbidity burden among SGLT-2i users, including more frequent CHF and more advanced DM as suggested by higher insulin use. However, in the propensity-matched cohort, SGLT-2i use was associated with lower in-hospital mortality, particularly in the CKD subgroup, in parallel with the reduction in AKI events. This finding supports the concept that nephroprotection may translate into improved early clinical outcomes in TAVI patients.

Most prior observational studies investigating SGLT-2i and AKI risk have focused primarily on diabetic populations (3, 12–16). However, SGLT-2i are not exclusively glucose-lowering agents; they also represent cornerstone therapies for cardiovascular disease, particularly in CHF (22–25). Furthermore, the nephroprotective effect of SGLT-2i has been shown to occur independent of DM status (7–11). In our study, 42.0% of SGLT-2i users were non-diabetic, reflecting contemporary real-world practice. Accordingly, matching and adjustment for DM and CHF were essential to achieve meaningful comparisons. Together, these results indicate that SGLT-2i may confer protection against AKI not only in diabetic patients but also across a real-world, heterogeneous TAVI population.

Although diabetes mellitus is a well-recognized risk factor for peri-procedural acute kidney injury, DM itself was not significantly associated with AKI in our cohort, whereas insulin use demonstrated only a borderline association on univariate analysis. In the overall population, insulin use was entered into the multivariable model but did not remain an independent predictor after adjustment for baseline renal function and other covariates. In the propensity score-matched cohort, insulin use was not included in the multivariable model because of the limited number of AKI events and the need to avoid model overfitting. These findings may reflect the fact that, in contemporary TAVI populations, the renal risk attributed to diabetes is often mediated through baseline renal dysfunction and overall comorbidity burden rather than the presence of diabetes alone, with CKD and baseline creatinine consistently emerging as the strongest predictors of AKI after TAVI. In this context, insulin therapy may identify patients with more advanced or long-standing diabetes and greater systemic disease severity, serving as a marker of higher-risk clinical status rather than a direct causal factor. Prior studies in TAVI populations have similarly suggested that diabetes alone does not independently predict AKI after adjustment for baseline renal function and comorbidities (26, 27).

A potential concern is that many patients underwent diagnostic coronary angiography shortly before TAVI, which may have increased cumulative contrast exposure and influenced AKI risk. In our institutional protocol, coronary angiography was typically performed approximately 2 days prior to TAVI in all patients, regardless of SGLT-2 inhibitor use. Importantly, cumulative contrast volume, including contrast administered during the hospitalization period for coronary angiography/PCI and the TAVI procedure, was comparable between SGLT-2i users and non-users after propensity score matching, including within CKD subgroups. Moreover, total contrast volume was included in the multivariable models, and SGLT-2i use remained an independent predictor of AKI after adjustment. These findings suggest that the lower AKI rates observed among SGLT-2i users are unlikely to be explained by differences in contrast exposure and may instead reflect a potential nephroprotective effect. The mean contrast volume for TAVI procedures in the registry study including 7,112 patients reported by Gualano et al. (28) was 106.4 ± 55.2 mL, and whereas our study cohort showed a mean cumulative contrast volume of 141.13 ± 81.49 mL. The slightly higher contrast volume observed in our study reflects the reporting of cumulative contrast exposure throughout the hospitalization period in this real-world registry, in order to avoid potential residual confounding from unreported contrast use during non-TAVI procedures. Considering this difference, the contrast volume used specifically for the TAVI procedure appears consistent with contemporary practice. In addition, efforts were routinely made to minimize contrast use during both coronary angiography and TAVI procedures to reduce the risk of AKI.

In the Society of Thoracic Surgeons/American College of Cardiology Transcatheter Valve Therapy registry including 107,814 patients, the incidence of AKI following TAVI was 10.7% (29). The Magna Graecia TAVI registry reported AKI rates of up to 15.3% after TAVI, with an incidence of 19.9% in the CKD subgroup (30). In our propensity-matched cohort, the overall AKI rate was 12.3%, with a rate of 23.8% in the CKD subgroup. These values are consistent with contemporary real-world TAVI registries, particularly given the high-risk profile and substantial CKD burden of our study population. Overall, these findings indicate that the AKI rates observed in our study are in line with contemporary TAVI practice and reflect a real-world population, thereby supporting the validity and robustness of our results.

Risk factors for AKI in patients undergoing TAVI are well established, and predictors such as baseline renal function, contrast volume, older age, and anemia have consistently been associated with AKI in this population. Beyond this prior knowledge, our study demonstrated that SGLT-2i use independently predicted AKI following TAVI after adjustment for these well-established risk factors. Therefore, SGLT-2i use may represent an additional clinically relevant factor for AKI risk stratification and prevention in contemporary TAVI practice, particularly among patients with CKD.

In addition to the potential nephroprotective benefit, the safety profile of SGLT-2 inhibitor therapy in the peri-procedural TAVI setting warrants careful consideration. In our propensity-matched cohort, a higher incidence of new permanent pacemaker implantation was observed among SGLT-2i users, and a higher rate of minor bleeding was noted in the non-CKD subgroup. However, these findings should be interpreted with caution. The absolute number of events was small, and subgroup analyses were limited by sample size, increasing the likelihood of chance findings. At present, there is no clear biologically plausible mechanism linking SGLT-2i therapy to conduction disturbances requiring pacemaker implantation or to an increased risk of minor bleeding in the TAVI setting. Moreover, no consistent differences were observed in major bleeding, major vascular complications, stroke, or overall procedural complications across the overall population or CKD-stratified cohorts, suggesting that these isolated differences likely reflect statistical variability rather than a true treatment-related signal. Notably, no cases of euglycemic ketoacidosis or severe metabolic complications were observed. Taken together, these findings suggest that continuation of SGLT-2i therapy during the hospitalization period was not associated with an excess of major procedural or metabolic adverse events. However, given the observational design and limited number of events, larger prospective studies are needed to further clarify the safety profile of peri-procedural SGLT-2i use in patients undergoing TAVI.

## Conclusion

In this single-center registry of patients undergoing transfemoral TAVI, SGLT-2 inhibitor use was associated with a significantly lower risk of post-procedural acute kidney injury and reduced need for hemodialysis, particularly among patients with chronic kidney disease. These findings remained consistent after propensity score matching and multivariable adjustment, in which SGLT-2i use independently predicted lower AKI risk. Our results suggest that SGLT-2 inhibitors may represent a clinically relevant strategy for peri-procedural renal protection and risk reduction in contemporary TAVI practice, especially in patients with baseline renal impairment.

## Limitations

This study has several limitations. First, this was a single-center observational analysis; therefore, residual confounding cannot be fully excluded despite propensity score matching and multivariable adjustment. Second, SGLT-2i use was not randomized, and unmeasured factors related to treatment indication and peri-procedural management may have influenced outcomes. Finally, the relatively limited number of AKI-related events, particularly in subgroup analyses, may have reduced statistical power, and long-term renal outcomes were not evaluated.

## Data Availability

Data supporting the results of this work can be obtained from the corresponding author upon reasonable request.
