# Supplementary material for: Sodium–glucose cotransporter-2 inhibitor use is associated with reduced acute kidney injury after transcatheter aortic valve implantation
Source: Front Cardiovasc Med. 2026 Mar 27;13:1799940. doi: 10.3389/fcvm.2026.1799940 (PMC13065653; doi:10.3389/fcvm.2026.1799940)
Supplement: Supplementary file 1 [file Datasheet1.docx]

Supplementary Material

# Supplementary Tables

**Supplementary Table 1. Comparison of Baseline Characteristics, Comorbidities, and Medications in Overall Patient Population**

| **Variables** | **Overall patient cohort**  **(n= 532)** | | | | **Non-CKD subgroup**  **(n=348)** | | | **CKD subgroup**  **(n=184)** | | |
| --- | --- | --- | --- | --- | --- | --- | --- | --- | --- | --- |
|  | **SGLT-2i user**  **(n= 112)** | **SGLT-2i non-user**  **(n= 420)** | | **p-value** | **SGLT-2i user**  **(n=68)** | **SGLT-2i non-user**  **(n=280)** | **p-value** | **SGLT-2i user**  **(n=44)** | **SGLT-2i non-user**  **(n=140)** | **p-value** |
| **Baseline Characteristics & Demographics** | | | | | | | | | | |
| **Age (years)** | 75.76 ± 8.54 | 78.22 ± 7.88 | 0.007 | | 75.37 ± 7.64 | 77.36 ± 8.02 | 0.051 | 76.36 ± 9.82 | 79.94 ± 7.31 | 0.040 |
| **Male sex** | 70 (62.5%) | 200 (47.6%) | 0.007 | | 45 (66.2%) | 124 (44.3%) | 0.002 | 25 (56.8%) | 76 (54.3%) | 0.863 |
| **BMI (kg/m^2^)** | 27.10 ± 5.25 | 27.71 ± 4.81 | 0.183 | | 26.51 ± 5.11 | 27.65 ± 4.67 | 0.033 | 28.06 ± 5.41 | 27.83 ± 5.10 | 0.678 |
| **Comorbidities** | | | | | | | | | | |
| **Hypertension** | 91 (81.2%) | 370 (88.1%) | 0.082 | | 59 (86.8%) | 243 (86.8%) | 1.000 | 32 (72.7%) | 127 (90.7%) | 0.005 |
| **Diabetes mellitus** | 65 (58.0%) | 109 (26.0%) | <0.001 | | 39 (57.4%) | 64 (22.9%) | <0.001 | 26 (59.1%) | 45 (32.1%) | 0.002 |
| **Atrial fibrillation** | 45 (40.2%) | 148 (35.2%) | 0.392 | | 23 (33.8%) | 91 (32.5%) | 0.886 | 22 (50.0%) | 57 (40.7%) | 0.299 |
| **Congestive heart failure** | 52 (46.4%) | 67 (16.0%) | <0.001 | | 31 (45.6%) | 27 (9.6%) | <0.001 | 21 (47.7%) | 40 (28.6%) | 0.030 |
| **COPD** | 61 (54.5%) | 188 (44.8%) | 0.085 | | 33 (48.5%) | 109 (38.9%) | 0.169 | 28 (63.6%) | 79 (56.4%) | 0.484 |
| **Previous stroke** | 2 (1.8%) | 20 (4.8%) | 0.192 | | 2 (2.9%) | 11 (3.9%) | 1.000 | 0 (0.0%) | 9 (6.4%) | 0.117 |
| **Chronic kidney disease** | 44 (39.3%) | 140 (33.3%) | 0.287 | | N/A | N/A | N/A | N/A | N/A | N/A |
| **Coronary artery disease** | 75 (67.0%) | 261 (62.1%) | 0.407 | | 45 (66.2%) | 167 (59.6%) | 0.336 | 30 (68.2%) | 94 (67.1%) | 1.000 |
| **Previous CABG** | 22 (19.6%) | 53 (12.6%) | 0.081 | | 18 (26.5%) | 30 (10.7%) | 0.002 | 4 (9.1%) | 23 (16.4%) | 0.329 |
| **Pre-operative pacemaker** | 3 (2.7%) | 18 (4.3%) | 0.589 | | 2 (2.9%) | 12 (4.3%) | 1.000 | 1 (2.3%) | 6 (4.3%) | 1.000 |
| **Medications** | | | | | | | | | | |
| **ACEi/ARB** | 63 (56.2%) | 249 (59.3%) | 0.637 | | 43 (63.2%) | 174 (62.1%) | 0.890 | 20 (45.5%) | 75 (53.6%) | 0.390 |
| **ß-blocker** | 104 (92.9%) | 360 (85.7%) | 0.064 | | 63 (92.6%) | 232 (82.9%) | 0.058 | 41 (93.2%) | 128 (91.4%) | 1.000 |
| **Statins** | 80 (71.4%) | 260 (61.9%) | 0.079 | | 49 (72.1%) | 175 (62.5%) | 0.159 | 31 (70.5%) | 85 (60.7%) | 0.285 |
| **Insulin** | 25 (22.3%) | 50 (11.9%) | 0.008 | | 10 (14.7%) | 27 (9.6%) | 0.271 | 15 (34.1%) | 23 (16.4%) | 0.018 |
| **Anticoagulation** | 40 (35.7%) | 146 (34.8%) | 0.939 | | 20 (29.4%) | 92 (32.9%) | 0.665 | 20 (45.5%) | 54 (38.6%) | 0.482 |

**Abbreviations:** CKD: Chronic kidney disease, SGLT-2i: Sodium-glucose cotransporter-2 inhibitor, BMI: Body-mass index, COPD: Chronic obstructive pulmonary disease, CABG: Coronary artery bypass grafting, ACEi: Angiotensin-converting enzyme inhibitor, ARB: Angiotensin receptor blockers.

**Supplementary Table 2. Comparison of Pre-Procedural Echocardiographic Parameters, Laboratory Values, and Procedural Data in Overall Patient Population**

| **Variables** | **Overall patient cohort**  **(n= 532)** | | | **Non-CKD subgroup**  **(n=348)** | | | **CKD subgroup**  **(n=184)** | | |
| --- | --- | --- | --- | --- | --- | --- | --- | --- | --- |
|  | **SGLT-2i user**  **(n= 112)** | **SGLT-2i non-user**  **(n= 420)** | **p-value** | **SGLT-2i user**  **(n=68)** | **SGLT-2i non-user**  **(n=280)** | **p-value** | **SGLT-2i user**  **(n=44)** | **SGLT-2i non-user**  **(n=140)** | **p-value** |
| **Pre-procedural echocardiographic parameters** | | | | | | | | | |
| **LVEF (%)** | 43.97 ± 13.00 | 52.95 ± 11.45 | <0.001 | 44.65 ± 12.29 | 55.00 ± 9.78 | <0.001 | 42.93 ± 14.10 | 48.86 ± 13.33 | 0.013 |
| **LVEDD (cm)** | 5.27 ± 0.66 | 4.86 ± 0.57 | <0.001 | 5.21 ± 0.58 | 4.78 ± 0.50 | <0.001 | 5.36 ± 0.77 | 5.03 ± 0.65 | 0.015 |
| **LVESD (cm)** | 3.81 ± 0.80 | 3.33 ± 0.68 | <0.001 | 3.72 ± 0.74 | 3.23 ± 0.57 | <0.001 | 3.94 ± 0.87 | 3.54 ± 0.84 | 0.013 |
| **Maximum aortic gradient (mmHg)** | 62.78 ± 23.74 | 72.37 ± 21.33 | <0.001 | 66.76 ± 23.51 | 74.34 ± 21.69 | 0.034 | 56.52 ± 22.98 | 68.48 ± 20.11 | 0.004 |
| **Aortic valve area (cm^2^)** | 0.74 ± 0.16 | 0.72 ± 0.16 | 0.296 | 0.73 ± 0.16 | 0.72 ± 0.16 | 0.583 | 0.76 ± 0.16 | 0.73 ± 0.15 | 0.355 |
| **Low-flow low-gradient AS** | 27 (24.1%) | 45 (10.8%) | <0.001 | 11 (16.2%) | 19 (6.9%) | 0.027 | 16 (36.4%) | 26 (18.7%) | 0.023 |
| **TAPSE (cm)** | 1.83 ± 0.31 | 1.95 ± 0.31 | 0.006 | 1.84 ± 0.33 | 1.99 ± 0.30 | 0.008 | 1.82 ± 0.29 | 1.86 ± 0.30 | 0.523 |
| **PASP (mmHg)** | 49.83 ± 14.63 | 49.17 ± 14.11 | 0.941 | 51.01 ± 15.07 | 47.85 ± 14.45 | 0.286 | 47.95 ± 13.88 | 51.82 ± 13.06 | 0.111 |
| **Left atrial diameter (cm)** | 4.50 ± 0.57 | 4.32 ± 0.58 | 0.004 | 4.49 ± 0.56 | 4.25 ± 0.55 | 0.003 | 4.51 ± 0.58 | 4.46 ± 0.63 | 0.470 |
| **Aortic root diameter (cm)** | 2.83 ± 0.51 | 2.70 ± 0.54 | 0.019 | 2.81 ± 0.50 | 2.70 ± 0.58 | 0.097 | 2.85 ± 0.52 | 2.71 ± 0.44 | 0.110 |
| **Moderate-to-severe mitral regurgitation** | 71 (63.4%) | 261 (62.1%) | 0.894 | 39 (57.4%) | 165 (58.9%) | 0.921 | 32 (72.7%) | 96 (68.6%) | 0.738 |
| **Moderate-to-severe tricuspid regurgitation** | 71 (63.4%) | 235 (56.0%) | 0.191 | 43 (63.2%) | 150 (53.6%) | 0.193 | 28 (63.6%) | 85 (60.7%) | 0.865 |
| **Moderate-to-severe aortic regurgitation** | 50 (44.6%) | 181 (43.1%) | 0.852 | 29 (42.6%) | 118/280 (42.1%) | 1.000 | 21 (47.7%) | 63 (45.0%) | 0.886 |
| **Laboratory values** | | | | | | | | | |
| **Pre-op hemoglobin (g/dL)** | 11.73 ± 1.71 | 11.53 ± 1.74 | 0.229 | 11.87 ± 1.75 | 11.88 ± 1.68 | 0.972 | 11.51 ± 1.63 | 10.82 ± 1.64 | 0.015 |
| **Pre-op creatinine (mg/dL)** | 1.20 ± 0.53 | 1.11 ± 0.50 | 0.030 | 0.91 ± 0.17 | 0.86 ± 0.19 | 0.026 | 1.64 ± 0.58 | 1.63 ± 0.54 | 0.717 |
| **Post-op hemoglobin (g/dL)** | 10.37 ± 1.59 | 10.13 ± 1.67 | 0.121 | 10.42 ± 1.49 | 10.31 ± 1.69 | 0.588 | 10.30 ± 1.74 | 9.77 ± 1.58 | 0.064 |
| **Leucocytes (cell count/L)** | (7.85 ± 2.25) x 10^3^ | (7.66 ± 2.65) x 10^3^ | 0.150 | (7.60 ± 2.14) x 10^3^ | (7.39 ± 2.24) x 10^3^ | 0.341 | (8.23 ± 2.38) x 10^3^ | (8.22 ± 3.26) x 10^3^ | 0.449 |
| **Platelets (cell count/mcL)** | (220.65 ± 64.87)x10^3^ | (227.93 ± 81.33)x10^3^ | 0.618 | (219.37 ± 64.84) x 10^3^ | (233.53 ± 84.45) x 10^3^ | 0.226 | (222.64 ± 65.61) x 10^3^ | (216.69 ± 73.70) x 10^3^ | 0.326 |
| **Sodium (mEq/L)** | 137.36 ± 3.42 | 137.96 ± 3.51 | 0.094 | 137.79 ± 3.34 | 138.37 ± 2.98 | 0.224 | 136.70 ± 3.47 | 137.09 ± 4.32 | 0.550 |
| **Potassium (mEq/L)** | 4.35 ± 0.52 | 4.33 ± 0.47 | 0.773 | 4.34 ± 0.44 | 4.30 ± 0.42 | 0.508 | 4.37 ± 0.63 | 4.41 ± 0.55 | 0.701 |
| **AST (IU/L)** | 37.52 ± 78.25 | 24.28 ± 14.70 | 0.384 | 26.56 ± 26.86 | 24.33 ± 12.41 | 0.963 | 54.63 ± 119.55 | 24.19 ± 18.52 | 0.107 |
| **TSH (mIU/L)** | 2.34 ± 2.65 | 2.01 ± 1.60 | 0.964 | 2.48 ± 3.15 | 1.93 ± 1.53 | 0.845 | 2.09 ± 1.46 | 2.18 ± 1.73 | 0.990 |
| **Procedural data** | | | | | | | | | |
| **Self-expanding platform use** | 84 (75.0%) | 289 (68.8%) | 0.245 | 51 (75.0%) | 200 (71.4%) | 0.652 | 33 (75.0%) | 89 (63.6%) | 0.201 |
| **Valve-in-valve procedure** | 8 (7.1%) | 18 (4.3%) | 0.220 | 7 (10.3%) | 13 (4.6%) | 0.083 | 1 (2.3%) | 5 (3.6%) | 1.000 |
| **Post-dilatation** | 31 (27.7%) | 118 (28.1%) | 1.000 | 19 (27.9%) | 79 (28.2%) | 1.000 | 12 (27.3%) | 39 (27.9%) | 1.000 |
| **Valve size (mm)** | 29.08 ± 3.62 | 27.96 ± 3.27 | 0.005 | 29.16 ± 3.75 | 27.80 ± 3.23 | 0.006 | 28.94 ± 3.46 | 28.27 ± 3.35 | 0.372 |
| **Total contrast volume (mL)** | 141.75 ± 82.63 | 123.78 ± 72.78 | 0.028 | 147.70 ± 80.03 | 122.05 ± 72.06 | 0.006 | 132.26 ± 86.74 | 127.30 ± 74.36 | 0.991 |

**Abbreviations:** CKD: Chronic kidney disease, SGLT-2i: Sodium-glucose cotransporter-2 inhibitor, LVEF: Left ventricular ejection fraction, LVEDD: Left ventricular end-diastolic diameter, LVESD: Left ventricular end-systolic diameter, AS: Aortic stenosis, TAPSE: Tricuspid annular plane systolic excursion, PASP: Pulmonary artery systolic pressure, AST: Aspartate aminotransferase, TSH: Thyroid-stimulating hormone.

**Supplementary Table 3. Standardized mean differences (SMDs) of baseline covariates before and after propensity score matching**

| **Variable** | **SMD before** | **SMD after** |
| --- | --- | --- |
| **Age** | -0,299 | 0,102 |
| **Sex** | -0,301 | -0,018 |
| **Body-mass index** | -0,120 | -0,053 |
| **Chronic kidney disease** | 0,123 | 0 |
| **Pre-op left ventricular ejection fraction** | -0,732 | 0,078 |
| **Diabetes** | 0,685 | 0,109 |
| **Insulin** | 0,278 | 0,021 |
| **Pre-op creatinine** | 0,162 | 0,077 |
| **Congestive heart failure** | 0,697 | 0,037 |

**Supplementary Table 4. Procedural and Study Outcomes in Overall Patient Cohort**

| **Variables** | **Overall patient cohort**  **(n= 532)** | | | **Non-CKD subgroup**  **(n=348)** | | | **CKD subgroup**  **(n=184)** | | |
| --- | --- | --- | --- | --- | --- | --- | --- | --- | --- |
|  | **SGLT-2i user**  **(n= 112)** | **SGLT-2i non-user**  **(n= 420)** | **p-value** | **SGLT-2i user**  **(n=68)** | **SGLT-2i non-user**  **(n=280)** | **p-value** | **SGLT-2i user**  **(n=44)** | **SGLT-2i non-user**  **(n=140)** | **p-value** |
| **Procedural Complications & Outcomes** | | | | | | | | | |
| **Myocardial infarction/Coronary obstruction** | 1 (0.9%) | 2 (0.5%) | 0.509 | 1 (1.5%) | 1 (0.4%) | 0.353 | 0 (0.0%) | 1 (0.7%) | 1.000 |
| **Stroke** | 0 (0.0%) | 8 (1.9%) | 0.214 | 0 (0.0%) | 3 (1.1%) | 1.000 | 0 (0.0%) | 5 (3.6%) | 0.340 |
| **LV rupture/Cardiac tamponade** | 2 (1.8%) | 4 (1.0%) | 0.611 | 1 (1.5%) | 4 (1.4%) | 1.000 | 1 (2.3%) | 0 (0.0%) | 0.239 |
| **Major vascular complication** | 10 (8.9%) | 33 (7.9%) | 0.698 | 8 (11.8%) | 24 (8.6%) | 0.481 | 2 (4.5%) | 9 (6.4%) | 1.000 |
| **Minor vascular complication** | 16 (14.3%) | 41 (9.8%) | 0.172 | 11 (16.2%) | 27 (9.6%) | 0.131 | 5 (11.4%) | 14 (10.0%) | 0.780 |
| **Need for surgery due to peripheral complication** | 6 (5.4%) | 12 (2.9%) | 0.235 | 5 (7.4%) | 12 (4.3%) | 0.343 | 1 (2.3%) | 0 (0.0%) | 0.239 |
| **Major bleeding** | 12 (10.7%) | 37 (8.8%) | 0.581 | 8 (11.8%) | 25 (8.9%) | 0.490 | 4 (9.1%) | 12 (8.6%) | 1.000 |
| **Minor bleeding** | 20 (17.9%) | 62 (14.8%) | 0.461 | 14 (20.6%) | 38 (13.6%) | 0.183 | 6 (13.6%) | 24 (17.1%) | 0.648 |
| **Device embolization** | 1 (0.9%) | 7 (1.7%) | 0.941 | 0 (0.0%) | 7 (2.5%) | 0.353 | 1 (2.3%) | 0 (0.0%) | 0.239 |
| **New permanent pacemaker** | 8 (7.1%) | 22 (5.3%) | 0.489 | 3 (4.4%) | 16 (5.7%) | 1.000 | 5 (11.4%) | 6 (4.3%) | 0.137 |
| **Technical success** | 93 (83.0%) | 372 (88.6%) | 0.159 | 55 (80.9%) | 245 (87.5%) | 0.221 | 38 (86.4%) | 127 (90.7%) | 0.587 |
| **Moderate-to-severe paravalvular regurgitation** | 5 (4.6%) | 19 (4.8%) | 1.000 | 3 (4.5%) | 15 (5.6%) | 1.000 | 2 (4.8%) | 4 (3.2%) | 0.640 |
| **In-hospital Outcomes** | | | | | | | | | |
| **Hospitalization duration (days)** | 5.45 ± 2.94 | 5.33 ± 4.04 | 0.133 | 4.72 ± 2.25 | 4.74 ± 3.38 | 0.312 | 6.57 ± 3.51 | 6.51 ± 4.92 | 0.387 |
| **In-hospital mortality** | 2 (1.8%) | 15 (3.6%) | 0.545 | 1 (1.5%) | 5 (1.8%) | 1.000 | 1 (2.3%) | 10 (7.1%) | 0.464 |
| **Study Outcomes** | | | | | | | | | |
| **Post-procedural acute kidney injury** | 5 (4.5%) | 67 (16.0%) | <0.001 | 3 (4.4%) | 18 (6.4%) | 0.777 | 2 (4.5%) | 49 (35.0%) | <0.001 |
| **Need for hemodialysis** | 1 (0.9%) | 25 (6.0%) | 0.025 | 1 (1.5%) | 4 (1.4%) | 1.000 | 0 (0.0%) | 21 (15.0%) | 0.005 |

**Abbreviations:** CKD: Chronic kidney disease, SGLT-2i: Sodium-glucose cotransporter-2 inhibitor, LV: Left ventricle.
